# Roles of the cerebellar vermis in predictive postural controls against external disturbances

**DOI:** 10.1038/s41598-024-53186-x

**Published:** 2024-02-07

**Authors:** Akira Konosu, Yuma Matsuki, Kaito Fukuhara, Tetsuro Funato, Dai Yanagihara

**Affiliations:** 1 Department of Mechanical Engineering and Intelligent Systems, The University of Electro-Communications, 1-5-1 Chofugaoka, Chofu, Tokyo 182-8585 Japan; 2 Department of Life Sciences, The University of Tokyo, 3-8-1 Komaba, Meguro-ku, Tokyo 153-8902 Japan

**Keywords:** Motor control, Cerebellum

## Abstract

The central nervous system predictively controls posture against external disturbances; however, the detailed mechanisms remain unclear. We tested the hypothesis that the cerebellar vermis plays a substantial role in acquiring predictive postural control by using a standing task with floor disturbances in rats. The intact, lesioned, and sham groups of rats sequentially underwent 70 conditioned floor-tilting trials, and kinematics were recorded. Six days before these recordings, only the lesion group underwent focal suction surgery targeting vermal lobules IV–VIII. In the naïve stage of the sequential trials, the upright postures and fluctuations due to the disturbance were mostly consistent among the groups. Although the pattern of decrease in postural fluctuation due to learning corresponded among the groups, the learning rate estimated from the lumbar displacement was significantly lower in the lesion group than in the intact and sham groups. These results suggest that the cerebellar vermis contributes to predictive postural controls.

## Introduction

In daily activities and sports, the central nervous system must predictively control the entire body to overcome transmission delays from sensory inputs to motor outputs^1^. Predictive control is especially important for postural stability, which requires high-dimensional processing to coordinate joints under gravity or physical disturbances. Typical expressions of predictive postural control include counteracting movements and adjustments of reactive gains against external or internal disturbances^2,3^.

Predictive controls require internal models, namely information on the correspondence between motor outputs and the resulting changes in the external environment or body state^4,5^. Synaptic plasticity between parallel fibers and Purkinje cells in the cerebellar cortex is thought to be the major site for installing internal models based on error signals and generating predictive motor commands during interactions with other brain regions^1,3^. Studies on voluntary movements, such as standing on tiptoes, lifting a part of the body, and transferring loads between limbs, have shown that cerebellar lesions impede the preparatory adjustments for canceling the internal postural sways or make them imprecise^6–8^. In addition, cerebellar lesions impair the adjustment of preparatory activities and reflex gains in the leg muscles against floor disturbances during standing tasks^9,10^.

In recent years, the neural mechanisms of predictive control have been vigorously investigated in animal experiments, focusing on the roles of each brain region and communication between regions^11–13^. However, as most of these studies used behavioral tasks that could be performed under head-fixed conditions, such as blinking, saccades, and licking, the detailed mechanisms of predictive postural control remain unclear. Under these circumstances, an experimental system for floor tilting, which has been widely utilized to evaluate postural stability^14^, has recently been developed for rats^15^. Biomechanical fluctuations in upright-standing rats due to the disturbance generally agreed with those in humans, and comparisons between the experimental data and a simulation suggested that the rats acquired predictive muscle outputs at the hind limb joints based on the time difference between the conditioning stimulus and disturbance.

The first step in neurophysiological examination of this rat system will be to identify the cerebellar region that plays a substantial role in embedding internal models for predictive postural control. Moreover, it will inform the targets of neural recordings, administration of medicines, and remedies, such as deep brain stimulation, for patients with ataxia. The cerebellar vermis projects to the reticulospinal and vestibulospinal tracts through the fastigial nuclei, and stimulation of these tracts has been shown to increase postural muscle tone in cats while standing^16^. Researches on cerebellar patients and animal disease models have revealed that lesions in the vermis or fastigial nuclei are associated with postural deficits^17–19^. Furthermore, it has been demonstrated that these regions specifically activate during the execution of postural tasks^20^. Cerebellar lobules IV–VIII form closed loops with the primary motor cortex^21,22^, and in particular, vermal lobules V–VIII receive dense projections from the motor area innervating the trunk and proximal limbs^23^. Given this information, it is suggested that vermal lobules IV–VIII play a central role in the embedding of internal models for predictive postural control, and that lesions in this region can result in deficits in acquiring predictive controls.

In this study, we tested the hypothesis that vermal lobules IV–VIII play substantial roles in acquiring the predictive postural controls, using the external disturbance task in rats.

## Results

### Lesioned area in the cerebellum

**Figure 1:**
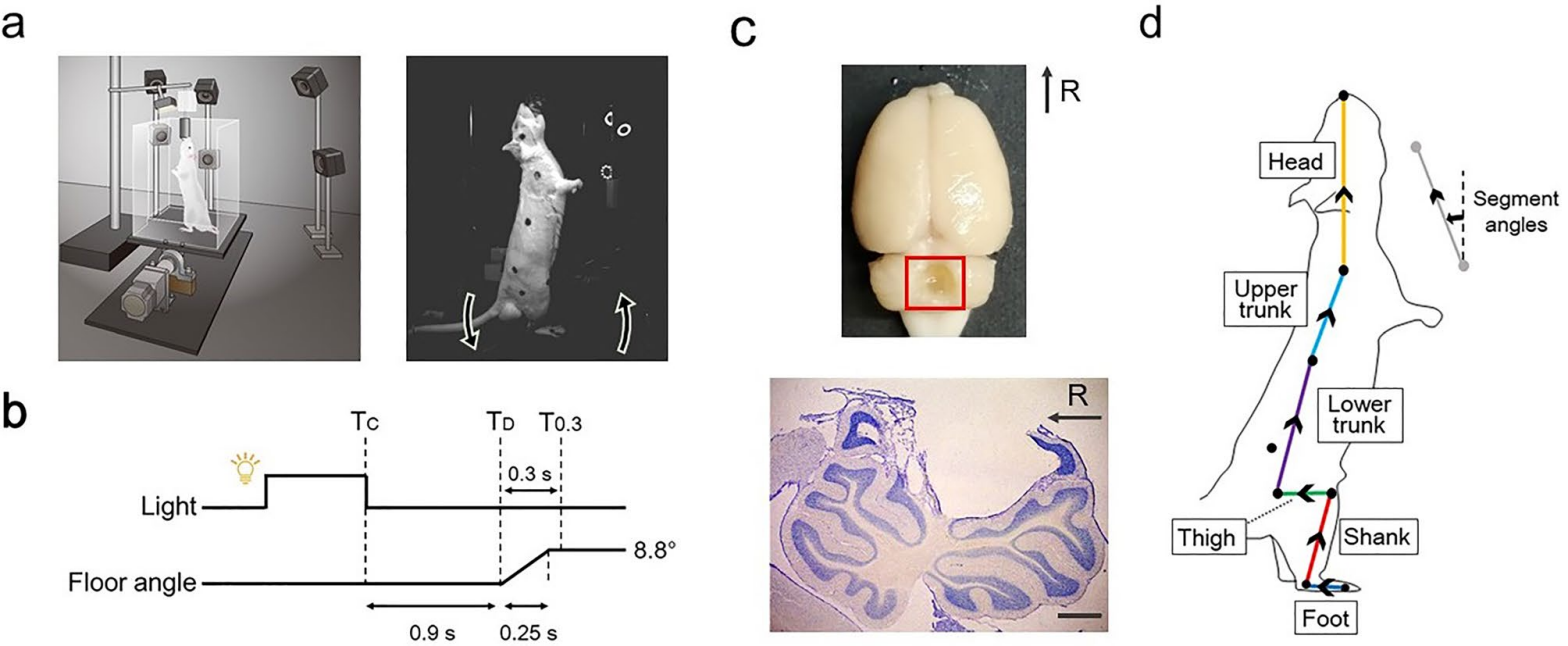
Experimental and analysis methods. (**a**) Experimental apparatus and rotation direction of the floor. (**b**) The paradigm of floor tilting trial. (**c**) Lesioned cerebellar area in a representative rat. Overall view of the brain (top) and the sagittal section of the cerebellum at the midline (bottom). R: rostral, scale bar: 1 mm. (**d**) Definitions of body segments and their angles.

**Figure 2:**
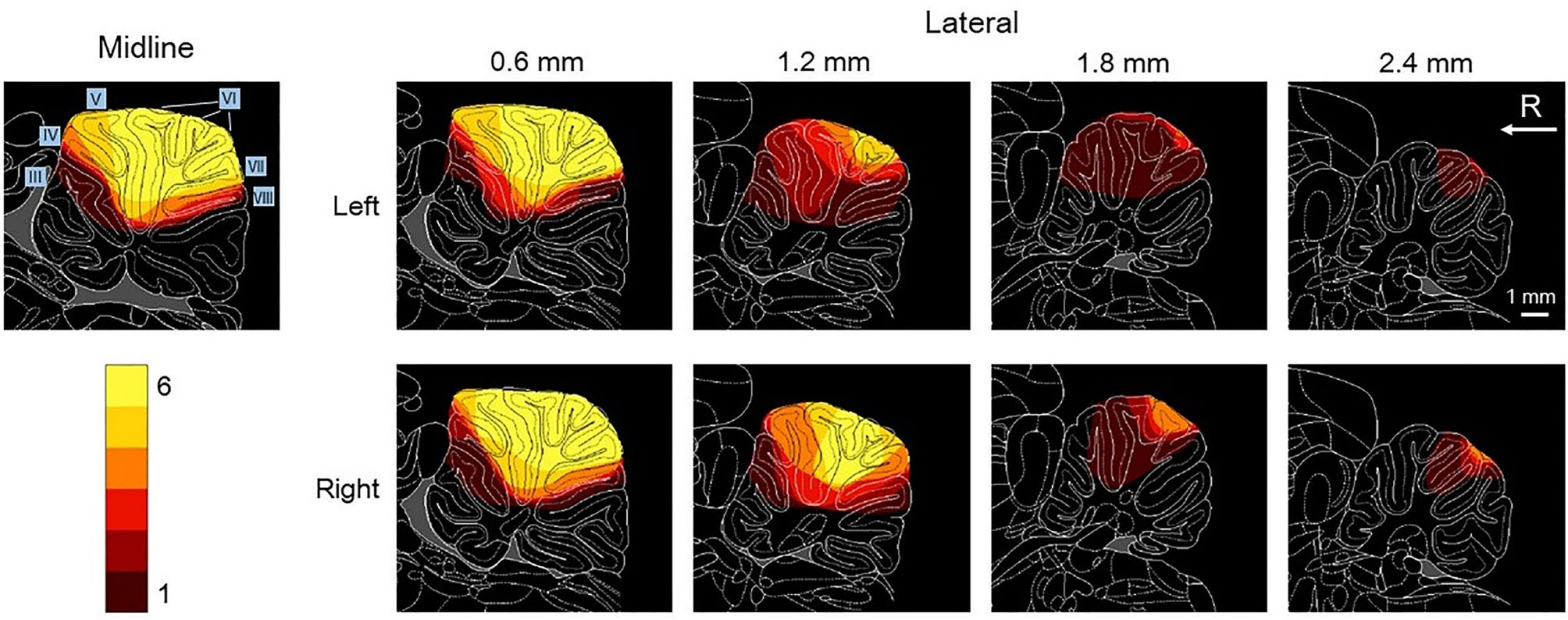
The lesioned area is represented on the rat atlas^37^, with the number of rats color-coded. R: rostral.

In the lesion group, the left and right extremities of the suctioned area were 1.9 ± 0.5 mm and 2.3 ± 0.6 mm lateral to the midline, respectively (Figs. 1c, 2, and S1). The rostral-caudal length of the area was 4.6 ± 0.2 mm, with lobules IV–VII lesioned in all six rats, lobule VIII lesioned in five rats, and lobule III lesioned in two rats. The opening area was 13.7 ± 2.1 mm^2^ and did not extend to the cerebellar nuclei.

### Postural fluctuation due to floor disturbance

**Figure 3:**
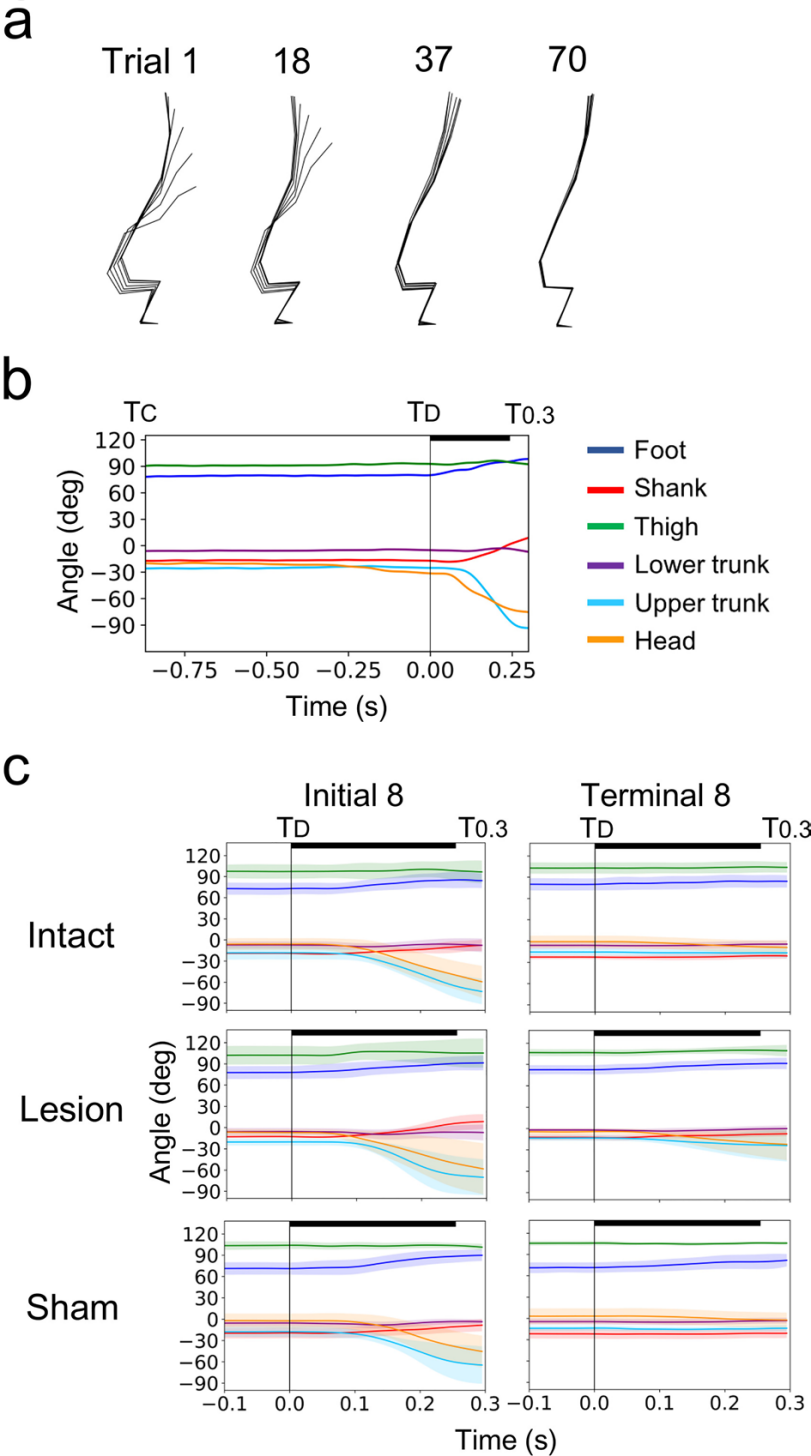
Postural fluctuations due to disturbance. (**a**) Stick pictures from the onset of floor disturbance to 0.2 s later. An example rat from the intact group is shown. (**b**) Time series of segment angles in a representative trial (trial 1 in figure a). (**c**) Group means and 95% confidence intervals of segment angles in the initial and terminal eight trials. The bold line indicates the floor tilting period. T_C_: conditioning stimulus, T_D_: onset of floor disturbance, T_0.3_: 0.3 s after the T_D_.

**Figure 4:**
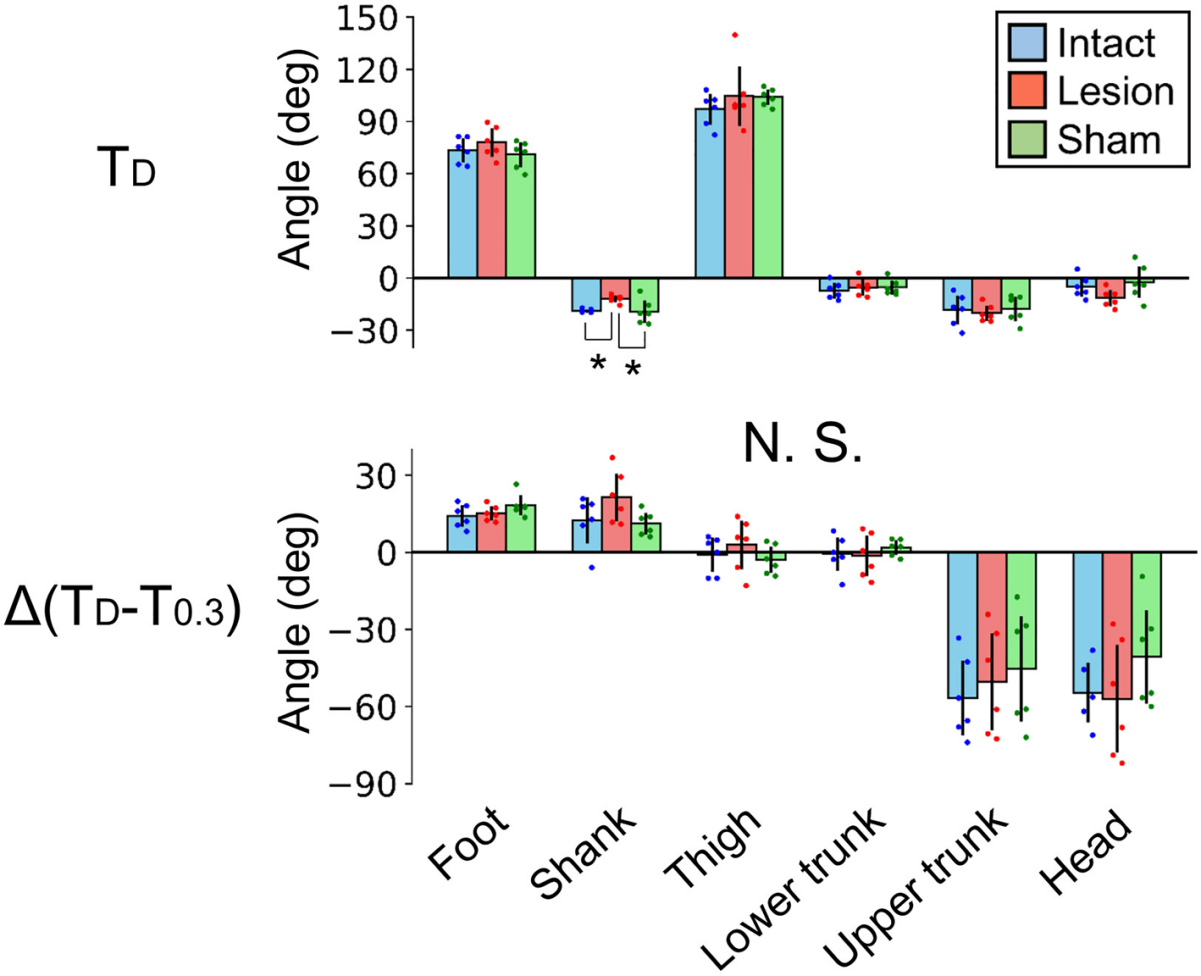
Posture and its fluctuation in the naïve stage. Segment angles at the onset of floor disturbance (T_D_) and rotation angles from T_D_ to 0.3 s later in the initial eight trials were compared between groups. Data from each rat (dot), as well as means and standard deviations are presented. *p < 0.05.

As motivation to remain standing, the rats were deprived of water for half a day before the measurements. On the measurement day, markers were applied under 2.5–3.0% isoflurane gas anesthesia, and after more than 30 min of awake time, they underwent sequential 70 floor tilting trials over 2–3 h. During the early stages of these trials, all three groups of rats swayed as they fell on the floor due to the disturbance (Fig. 3a). Specifically, upon the onset of floor tilting (T_D_), the foot and shank segments rotated backward while the upper trunk and head segments rotated forward in a monotonic fashion (Fig. 3b,c). To compare the group differences in posture and its fluctuation during this stage, we analyzed the averages of the initial eight trials in the segment angles at T_D_ and the segment rotations from T_D_ to 0.3 s later (T_0.3_) (Fig. 4). As a result, only the angle of the shank segment at T_D_ in the lesion group (− 12.0 ± 2.0°) was different from those in the intact (− 19.0 ± 1.0°, *p* = 0.031) and sham (− 19.3 ± 6.4°, *p* = 0.024) groups.

### Acquirement of predictive controls

**Figure 5:**
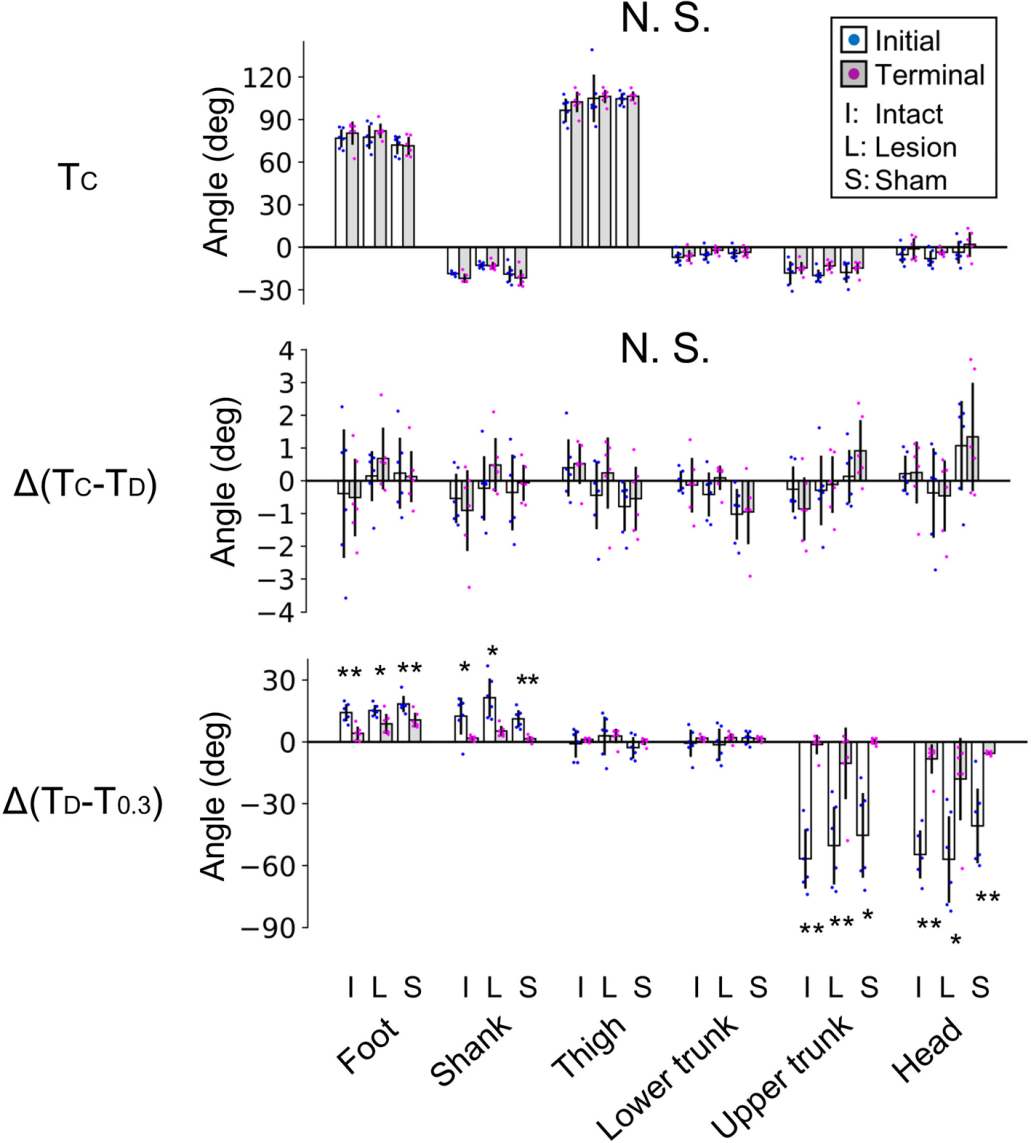
Changes in posture and its fluctuation due to learning. In each group, the segment angles at conditioning stimulus (T_c_) and rotation angles from T_C_ to the onset of floor disturbance (T_D_) and from T_D_ to 0.3 s later were compared between the initial and terminal eight trials. Data from each rat (dot), as well as means and standard deviations are presented. *p < 0.05, **p < 0.01.

In all groups, postural fluctuations due to disturbance decreased gradually through repeated trials (Fig. 3a,c). To investigate the changes in posture and fluctuations due to learning, we compared the segment angles and rotations between the initial eight and terminal eight trials in each group (Fig. 5). There were no differences in the segment angles at conditioning stimulus (T_C_) and the rotation angles from the T_C_ to T_D_ (*p* > 0.05). However, in all groups, the rotation angles from T_D_ to T_0.3_ reduced in the foot (*p* = 0.002, *p* = 0.033, and *p* = 0.009 in the intact, lesion, and sham groups, respectively), shank (p = 0.044, *p* = 0.011, and *p* = 0.003), upper trunk (*p* < 0.001, *p* = 0.006, and *p* = 0.004), and head (*p* = 0.020, *p* = 0.013, and *p* = 0.007) segments.

### Learning rate

**Figure 6:**
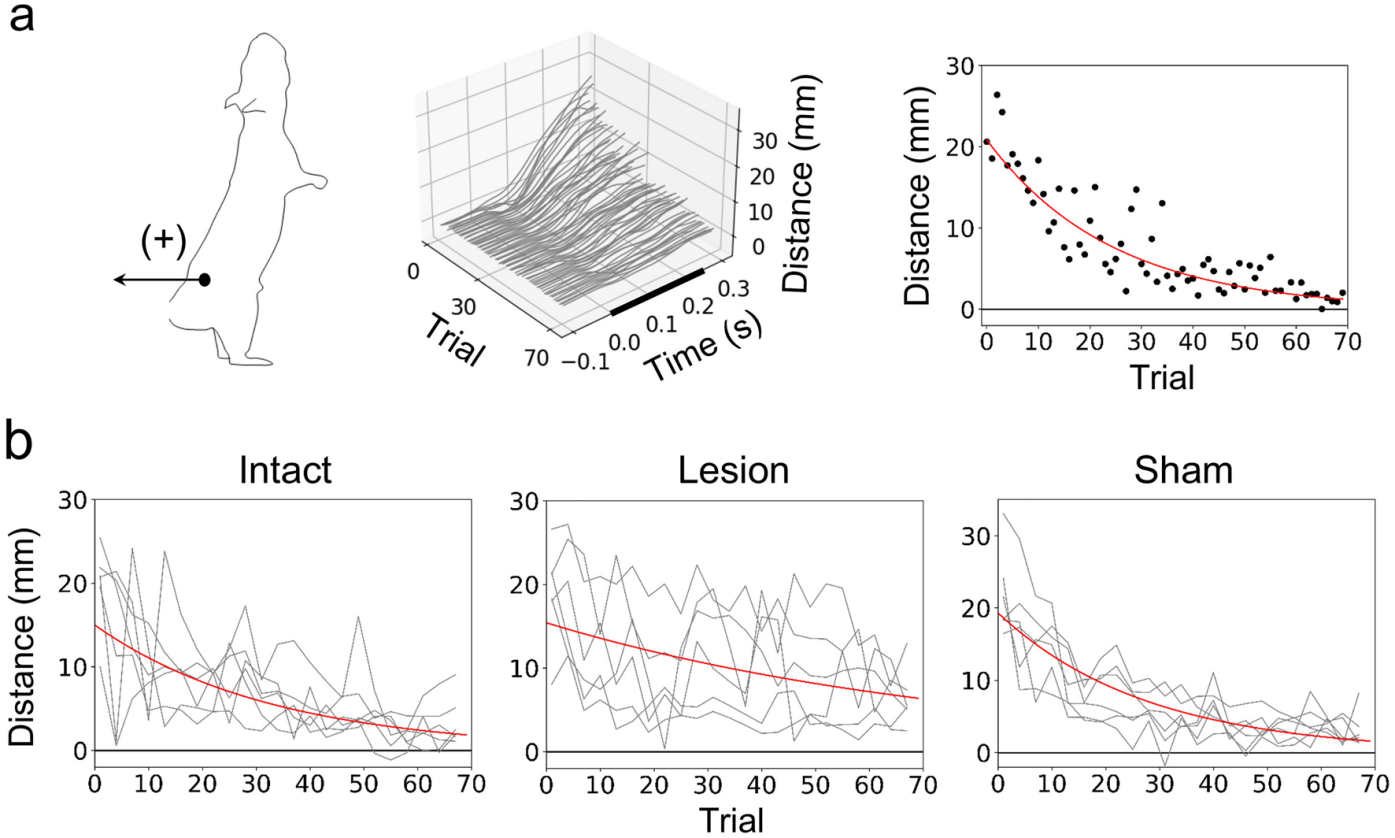
Evaluation of learning rate. (**a**) The horizontal position of the iliac crest marker relative to the onset of disturbance (time = 0), with the backward taken positive. The bold line indicates the floor tilting period. The first trial was set to 0. The learning rate was determined by exponential regression (Eq. 1) of the horizontal displacement of the marker from the onset of disturbance to 0.3 s later (right, red line). Data of a representative rat in the intact group is shown. (**b**) The same displacements as in the right of the figure (a) are shown for all rats in each group, with the averages of three peripheral trials plotted. The red lines were drawn by substituting the group averages of coefficients $A$ and ${{\text{log}}}_{10}B$ into Eq. (1).

**Figure 7:**
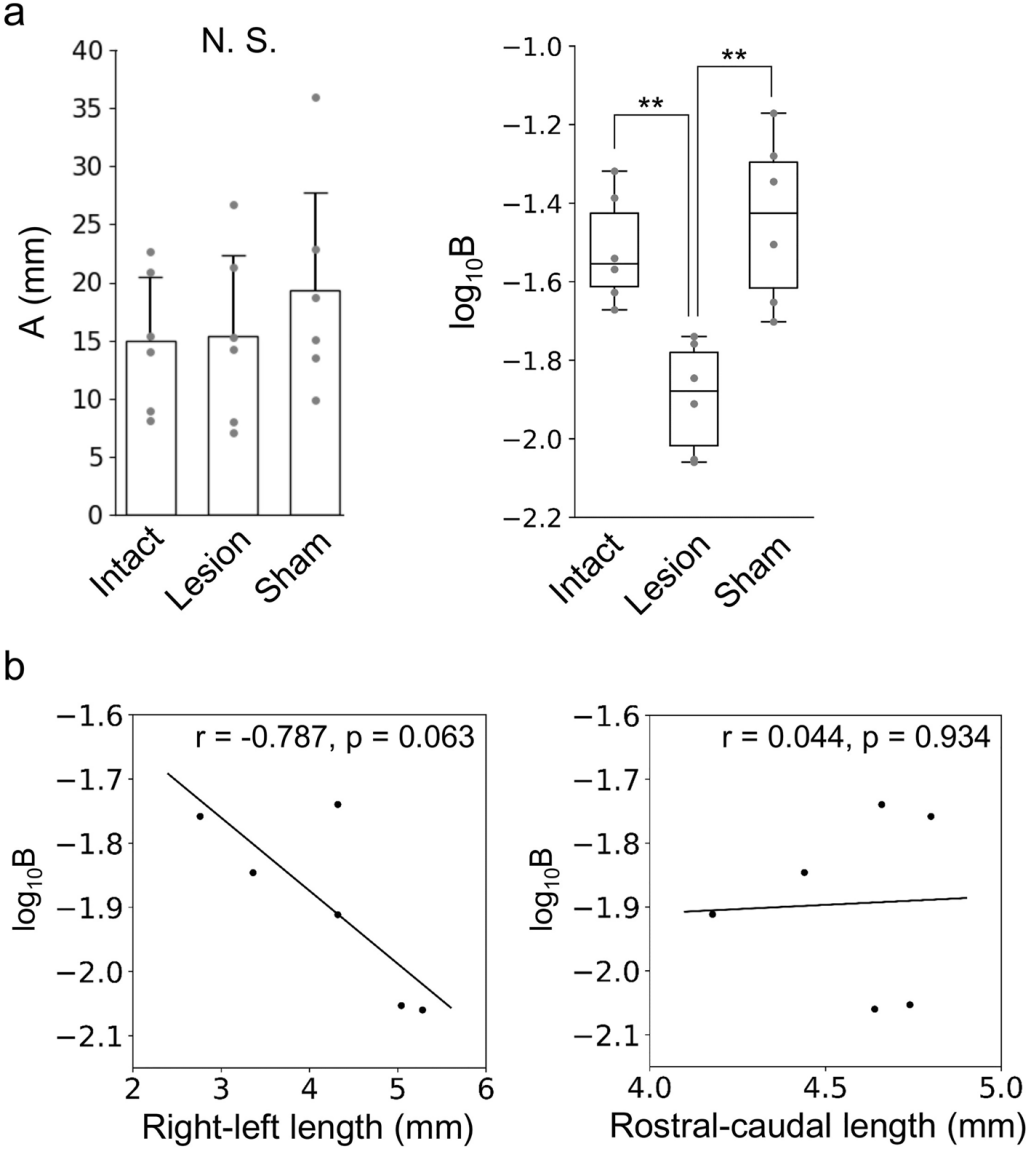
Coefficients in learning formula (Eq. 1). (**a**) Coefficient $A$ is shown as means and standard deviations of rats, and ${{\text{log}}}_{10}B$ (learning rate) is shown as boxplots. Data from each rat is dotted. **p < 0.01. (**b**) Relations between the sizes of lesioned area (right-left and rostral-caudal lengths of the opening area) and ${{\text{log}}}_{10}B$ were examined by Pearson’s correlation coefficient test.

To examine group differences in the reduction rates of postural fluctuation due to learning, we focused on the iliac crest marker, which showed the greatest backward displacement during the disturbance (Fig. 3a). As shown in Fig. 6, the horizontal displacement of the marker after T_D_ exponentially decreased as the number of trials increased. Therefore, we performed exponential regression on the displacement from T_D_ to T_0.3_ to calculate coefficients $A$ and $B$ (Eq. 1), which correspond to the initial displacement value and the learning rate, respectively (group-averaged determination coefficients R^2^ = 0.38, 0.18, and 0.54 in the intact, lesion, and sham groups, respectively). Although the coefficient $A$ was not different among the groups (*p* > 0.05), ${{\text{log}}}_{10}B$ was significantly smaller in the lesion group (− 1.89 ± 0.13) than in the intact (− 1.51 ± 0.13, p = 0.004) and sham (− 1.44 ± 0.19, *p* = 0.001) groups (Fig. 7a). According to the group averages of ${{\text{log}}}_{10}B$, the learning rate in the lesion group was 35% of that in the sham group.

The relationship between the size of the lesioned area and the learning rate was examined (Fig. 7b). Although the rostral-caudal length of the opening area showed no correlation with the learning rate (r = − 0.044, *p* = 0.934), the right-left length showed an almost significant negative correlation (r = − 0.787, *p* = 0.063).

## Discussion

In this study, our goal was to test the hypothesis that vermal lobules IV–VIII (target area) play a significant role in the acquisition of predictive postural controls. To achieve this, we performed suction surgeries on the target area and conducted experiments on rats using a disturbance task. The lesion was mostly confined to the target area (Fig. 2)^24^. In the naïve stage of 70 sequential trials, the standing posture and its fluctuation pattern in the lesion group did not differ from those in the intact and sham groups, except for a backward tilt of approximately 7° in the shank segment (Figs. 3c, 4 and 7a). This indicates that the lesion did not reduce the muscle strength required to maintain an upright posture against gravity or external disturbances. The pattern of decrease in postural fluctuation due to learning corresponded among the groups (Figs. 3c and 5); however, the learning rate estimated from the movement of the iliac crest was lower in the lesion group than in the intact and sham groups (Fig. 7a). In addition, the learning rate was dependent on lesion size (Fig. 7b). These results suggest that the target area contributes to the efficient learning of predictive postural controls against external disturbances, supporting our hypothesis.

The vermis can be divided into two zones, depending on the projection targets from the Purkinje cells^25^. These zones may play different roles in postural control: zone A projects to the reticular formation, lateral vestibular nuclei, and thalamus through the fastigial nuclei, suggesting an involvement of multiple CNS regions, including the cerebral cortex; zone B, located laterally, directly projects to the lateral vestibular nuclei, which contributes to balance control against support face disturbances with the activation of extensor muscles, and makes the co-activation timing of antagonist muscles accurate^26^. Zone A was lesioned in all rats, whereas zone B was lesioned in four rats (Fig. 2)^24^. Therefore, the learning rate seemed to reflect the sizes and proportions of the non-lesioned areas in both zones (Fig. 7b).

As previously discussed^15^, the basic mechanics of postural fluctuations due to floor-tilting disturbances are common between bipedal rats and humans. Therefore, the kinematic and neural strategies adopted by the rats in this study can be extrapolated from human studies. First, adjustments of reflex gains in ankle muscles are common strategies against floor disturbances in humans^9^, and the same adjustments around the metatarsophalangeal (MTP) and ankle joints seemed to have greatly contributed to the present experiment (Fig. 5). Secondly, a posterior displacement in the center of pressure and fluctuations in leg muscle activities are observed just before the disturbance in humans^10^, suggesting a strategy to reduce the difference in angular velocities between the floor and body or joint stiffness. Although the movement for drinking water and instability in bipedal rats (Fig. 3b) prevented the statistical detection of preparatory movements before T_D_ (Fig. 5), similar strategies might have contributed to the adaptation in the present experiment. Finally, in catch trials without disturbance, canceling movements during the timing of disturbance have been observed in the legs of humans^27^, and similar factors might have contributed to the present experiment. These studies demonstrated that cerebellar diseases impair the establishment of at least the former two strategies. Thus, our results suggest that the cerebellar vermis is a region that embeds internal models to execute these predictive strategies. We will validate this in the future by modeling and simulating studies of postural dynamics during learning.

Although the presence of a cerebellar lesion slowed the learning of predictive postural controls, it did not completely suppress learning (Figs. 6b and 7a). Several possible explanations may justify this finding. First, the part of the cerebellar area that contributes to predictive postural controls might have been unaffected by the lesion and continued driving learning. Because the right–left lesion length exhibited an almost significant negative correlation with learning rate (Fig. 7b), its additional expansion was expected to decrease the learning rate, eventually stopping the learning completely. In other words, the area involved in predictive postural controls was distributed laterally beyond the lesioned area. As mentioned above, the lesion was confined to zone A of the vermis in two rats but reached zone B in the other rats, suggesting that both zones contribute to learning. Fastigial nuclei, which were not lesioned in this study, may also drive learning. In our preliminary experiments, rats with lesions reaching the cerebellar nuclei were unable to stand on their hind limbs (Fig. S2). Because the projections from the cerebellar cortex aggregate at the nuclei, even a focal lesion in the nuclei seemed to severely impair postural function. Since this study focused only on the predictive part of postural controls, it was desirable that the standing posture itself and postural fluctuation due to disturbance at the naïve stage roughly corresponded between the intact and lesioned rats (Fig. 4). Therefore, we excluded the fastigial nuclei from the lesion targets. However, the fastigial nuclei may play important roles in the learning of predictive postural controls through extracerebellar transmission of activities in the vermis and plasticity^28^. Establishing a complete map of the cerebellar area contributing to predictive postural controls is an urgent issue. To clarify the detailed functions of each area, optimization of the experimental paradigms, including amplitude, angular velocity, and floor tilt direction, is necessary.

Second, neural reorganization is a possible cause of incomplete learning suppression. The cerebellum has a high ability for functional recovery owing to its abundant plasticity^29,30^, and it has been demonstrated that behavioral performance recovers remarkably within a few weeks after a cerebellar lesion in rats^30^. An fMRI study showed that the contralateral regions of the cerebellum and cerebral cortex were activated during recovery from cerebellar infarctions^31^. Thus, in the present study, the cerebrocerebellar loop might have been reorganized so that the cerebellar areas, which had originally been irrelevant to predictive postural controls, became responsible for those functions during the 6 days from surgery to measurements. These compensatory effects could be further investigated by conducting experiments with optogenetics or neural inhibitors to inhibit the target area and comparing the results to those obtained from the present data. This is a promising direction for our future research. If the cerebellum has a high capability for the reorganization of circuits for predictive postural control, elucidating its mechanisms will contribute to rehabilitation and drug discovery in patients with ataxia.

In recent years, studies on cerebrocerebellar interaction mechanisms for predictive control have been conducted using conditioning tasks in animal models. The key findings are as follows. First, increases in the activity of the anterior lateral motor region during the interval between the conditioning stimulus and motor outputs are substantial for predictive outputs, and concurrent cerebellar activities are critical for these activities^32,33^. Second, conditioned learning increases theta-band activity synchronized between the cerebral cortex and medial prefrontal cortex during the interval time, and the strength of its coherence correlates with the accuracy of motor commands and the learning rate^11^. Therefore, the circuit formed by the frontal cortex and vermis may generate and maintain predictive postural commands through theta-band communication. Our next goal is to elucidate the intricate mechanisms involved by recording neural activities during the disturbance task.

## Methods

### Experimental animals

Three groups of male Wistar rats (intact: n = 6, 19 ± 2 weeks old, 404 ± 28 g; lesion: n = 6, 22 ± 5 weeks old, 399 ± 18 g; sham: n = 6, 21 ± 1 weeks old, 418 ± 19 g) supplied by CLEA (Tokyo, Japan) were used for the experiment. The rats were housed in a room with a constant temperature and a 12-h light and dark cycle and had ad libitum access to food and water. The experiment was approved by the Ethics Committee for Animal Experiments at the University of Tokyo and was conducted in accordance with the Guidelines for Research with Experimental Animals of the University of Tokyo. This study was conducted and reported in accordance with the Animal Research: Reporting of In Vivo Experiments (ARRIVE) guidelines.

### Experimental protocols

The habituation and measurements were performed in a transparent box on a tilting plate in a dark room (Fig. 1a). During a period of 2–3 weeks of habituation prior to measurements, the rats were trained to maintain a standing posture on their hind limbs for more than 10 s to drink 4% sucrose water supplied from an overhead supply. Cerebellar surgeries were performed 6 days before the measurements in the lesion group. Specifically, a focal region in the cerebellar cortex targeting vermal lobules IV–VIII was suctioned under 3.0–5.0% isoflurane gas anesthesia (Figs. 1c and S1). In the sham group, a craniotomy was performed in the same cortical area.

Each rat underwent sequential 70-floor tilting trials in a day. The paradigm of each trial was as follows (Fig. 1b): First, the lights installed in front of the rats were turned on, and simultaneously sucrose water was supplied as a reward through a flexible tube suspended above the center of the floor. The rats stood upright on their hind limbs in the direction of the light to drink water. The measurer entered an electrical trigger into the system after confirming that the rat was in proper posture (the body was stationary, and the stomach was facing the light). This trigger turned the light off as a conditioning stimulus (T_C_), and the floor began to rotate backward as an external disturbance about 0.9 (0.91 ± 0.04) s later (T_D_). The floor was rotated by 8.8° over 0.25 s, with a constant angular velocity. The height of the water supply port was determined for each rat during the habituation so that the heels are not floated, and the hips do not touch the floor during the standing posture (intact group: 24.0 ± 0.3 cm; lesion group: 24.2 ± 0.4 cm; sham group: 24.0 ± 0.3 cm).

In the lesion group, perfusion fixation and sucrose replacement of the brain tissue were performed within 3 weeks of the measurements. The tissues were then sliced into sagittal sections of 40 μm in thickness and subjected to Nissl staining (Fig. 1c).

### Measurement of kinematics

Prior to the measurements, the right-sided body surface of the rats was shaved, and markers using black ink were applied to eight body landmarks (4th MTP joint, lateral malleolus, knee joint, greater trochanter, iliac crest, scapula, midpoint between the iliac crest and scapula, and temporomandibular joint) under anesthesia using 2.5–3.0% isoflurane gas. Six high-speed cameras (Prime 13 and 13 W; NaturalPoint Inc., United States) recorded the movements of the right side of the body at 200 Hz.

### Data processing

The two-dimensional coordinates of the markers and snout on the video images were determined using DeepLabCut^34^. The three-dimensional coordinates of each marker were calculated using the direct linear transformation method^35^. The coordinates were then smoothed using a 15 Hz 4th-order Butterworth low-pass filter. The angle of each segment was calculated using a rigid-body link model consisting of the foot, shank, thigh, lower trunk, upper trunk, and head segments (Fig. 1d).

To determine the rate of learning, the horizontal displacements of the iliac crest due to the disturbance versus trial numbers were regressed on the following exponential formula^36^ using the least-absolute method:

1 $$y=A{e}^{-Bx}$$

where $x$ is the trial number with the first being 0, and $y$ is the horizontal displacement of the iliac crest marker from the onset of floor tilting to 0.3 s later (posterior is taken positive). Coefficients $A$ and $B$ represent the expected displacement in the first trial and learning rate, respectively.

### Statistical analyses

The lesioned area and kinematic data are reported as mean ± standard deviation in each group. To examine group differences (intact: n = 6; lesion: n = 6; sham: n = 6) in posture and its fluctuation in the naïve stage, one-way analysis of variance (ANOVA) was performed on the averages of the angles and rotation angles of the segments in the initial eight trials, and the coefficient $A$ (Eq. 1). If significance was detected, a post-hoc comparison was performed using Tukey’s test. To examine the changes in posture and its fluctuation due to learning in each group (intact: n = 6; lesion: n = 6; sham: n = 6), the angles and rotation angles of the segments were compared between the initial and terminal eight trials using a two-sided Welch’s t-test. In these analyses, the head segment data of an intact rat after the onset of the floor disturbance in the initial eight trials were excluded because it tended to turn its head to the left in response to the disturbance. To examine the group differences (intact: n = 6; lesion: n = 6; sham: n = 6) in the learning rates, on a logarithm of coefficient $B$ (${{\text{log}}}_{10}B$), the normality and homogeneity of the variances were confirmed using the Shapiro–Wilk test and Bartlett’s test, respectively. One-way ANOVA was performed, and if significance was detected, post-hoc comparisons were made with Tukey’s test. Note that the variances of $B$ were judged to differ among the groups, whereas those of ${{\text{log}}}_{10}B$ were not. The relationship between the size of the lesioned area (right-left and rostral-caudal lengths of the opening area) and ${{\text{log}}}_{10}B$ was examined using Pearson’s correlation coefficient test. In the above tests, statistical significance was set at *p* < 0.05.

### Supplementary Information

Supplementary Figures.

## Data Availability

Upon acceptance of this paper, the trained DeepLabCut network, kinematic data, and analysis codes will be available at the Dryad data repository (10.5061/dryad.1vhhmgqzx).
