# Supplementary material for: Roles of the cerebellar vermis in predictive postural controls against external disturbances
Source: Sci Rep. 2024 Feb 7;14:3162. doi: 10.1038/s41598-024-53186-x (PMC10850480; doi:10.1038/s41598-024-53186-x)
Supplement: Supplementary file 1 — Supplementary Figures. [file 41598_2024_53186_MOESM1_ESM.docx]

Supplementary information

**Roles of the cerebellar vermis in predictive postural controls against external disturbances**

Akira Konosu, Yuma Matsuki, Kaito Fukuhara, Tetsuro Funato & Dai Yanagihara

**
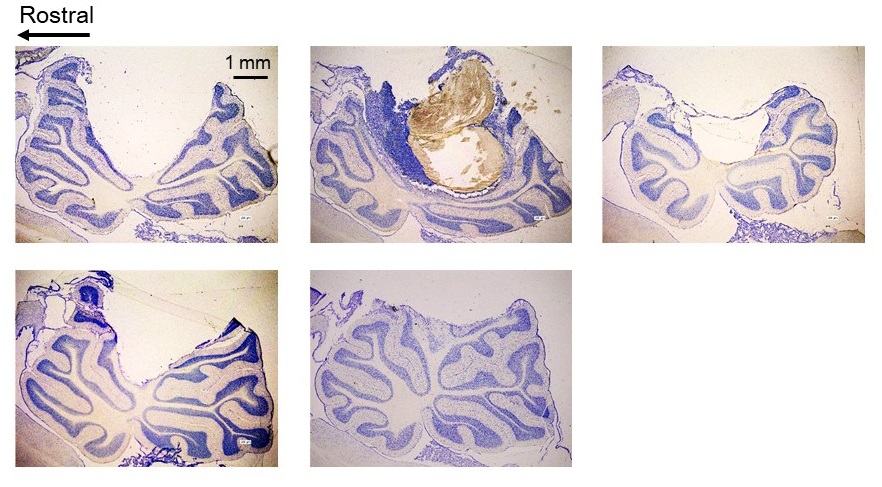
**

**Figure S1.** Sagittal sections (midlines) of the rats other than the one in Fig. 1c in the lesion group.


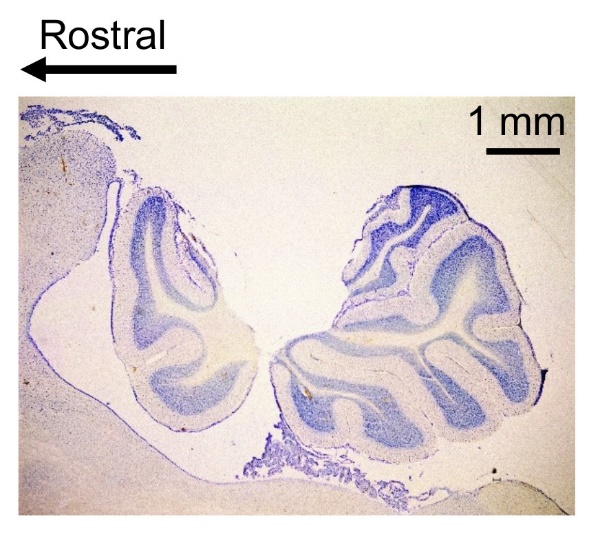


**Figure S2.** The same section as in Figs. 1c and S1 from a rat that was unable to stand on its hind limbs after the surgery. In addition to the target area, cerebellar nuclei had been lesioned.
